# Recent Trends and Effectiveness of Antiretroviral Regimens Among Men Who Have Sex With Men Living With HIV in the United States: The Multicenter AIDS Cohort Study (MACS) 2008–2017

**DOI:** 10.1093/ofid/ofz333

**Published:** 2019-07-16

**Authors:** Xiuhong Li, Todd T Brown, Kenneth S Ho, Mallory D Witt, John Phair, Lisa P Jacobson

**Affiliations:** 1 Department of Epidemiology, Johns Hopkins Bloomberg School of Public Health, Baltimore, Maryland; 2 Division of Endocrinology, Diabetes, and Metabolism, Johns Hopkins University School of Medicine, Baltimore, Maryland; 3 Department of Medicine, Division of Infectious Diseases, University of Pittsburgh School of Medicine, Pennsylvania; 4 Division of HIV Medicine, Los Angeles Biomedical Research Institute at Harbor-UCLA Medical Center, Torrance, California; 5 Department of Medicine, Division of Infectious Diseases, Northwestern University Feinberg School of Medicine, Chicago, Illinois

**Keywords:** cART, effectiveness, guidelines, IPTC weighted model, trend

## Abstract

**Objective:**

We evaluated trends and population effectiveness (tolerability, HIV suppression) of current combination antiretroviral therapy (cART) regimens mindful of treatment guidelines.

**Method:**

Trend analyses included 18 017 person-visits (1457 men) on cART during 2008–2017 in the Multicenter AIDS Cohort Study. Effectiveness analyses of current regimens used 3598 person-visit–pairs (745 men) on cART in 2014–2017. Inverse-probability-of-treatment-and-censoring weighted Poisson regression with robust variances was used to evaluate the association between regimens and switching, adherence and HIV RNA <20 copies/mL.

**Results:**

Integrase strand transfer inhibitor (INSTI)-based regimen usage has increased since 2008. Almost 90% of cART initiators started with INSTI-cART in 2016–2017; cART adherence was stable around 90% and 83%–85% suppressed virus (<20 cp/mL). Commonly used regimens in 2014–2017 contained disoproxil fumarate/emtricitabine (TDF/FTC) backbone with efavirenz (EFV, n = 1161 person-visits), elvitegravir/cobicistat (EVG/c, n = 551), rilpivirine (RPV, n = 492), darunavir/ritonavir (DRV/r, n = 351), or atazanavir (ATV)/r (n = 333). Others were dolutegravir/abacavir/lamivudine (DTG/ABC/3TC, n = 401) and EVG/c/tenofovir alafenamide/FTC (EVG/c/TAF/FTC, n = 309). Compared to EFV/TDF/FTC users, ATV/r+TDF/FTC users switched more (rate ratio [RR] = 1.80, 95% confidence interval (CI), 1.17–2.76), while those on DTG/ABC/3TC (RR [95% CI] = 0.16 [0.08–0.31]) or EVG/c/TAF/FTC (RR [95% CI] = 0.12 [0.06–0.27]) switched less. The rate of suppressed HIV RNA was 15% (95% CI, 2%–26%) lower among younger EVG/c/TDF/FTC users and 18% (95% CI, 3%–34%) higher in older DRV/r+TDF/FTC users; adherence did not differ by regimen.

**Conclusions:**

Consistent with guidelines, recent cART initiators started with INSTI-cART, which was associated with less switching early after initiation. Factors beyond those studied here, such as need for salvage therapy, unique personal characteristics, drug interactions, and cost may influence treatment decisions.

## Teaser

Consistent with guidelines, most recent cART initiators started with INSTI-cART, which was associated with less switching. Factors beyond those studied here, such as need for salvage therapy, unique personal characteristics, drug interactions, and cost may influence treatment decisions.

## INTRODUCTION

Combination antiretroviral therapy (cART) that contains 3 active drugs from 2 or more drug classes [1] has been available to treat HIV infection for 2 decades. Many studies have shown the effectiveness of cART for reducing HIV-related morbidity and mortality [2–7] by suppressing HIV and increasing CD4+ cell count [8–10]. Current cART regimens [11] do not eradicate HIV; restoring immunologic function and suppressing HIV RNA are primary treatment goals [1].

The emergence of integrase strand transfer inhibitors (INSTI) in 2007 and new multiclass single-pill drugs containing INSTI elvitegravir (EVG), bictegravir, or dolutegravir (DTG) after 2013 have broadened cART options. Reports on cART trends in the US in 2000-2008 [12] mainly assessed the patterns of protease inhibitor (PI) and nonnucleoside reverse transcriptase inhibitor (NNRTI) use among cART initiators. A recent publication showed that the percentage receiving an INSTI regimen increased from 10% to 40% between 2009 and 2016 in British Columbia’s Centre for Excellence in HIV/AIDS Drug Treatment Program in Canada [13]. Little is known about the uptake of INSTI-containing regimens in people with HIV in the United States. Although clinical trials demonstrated their comparable efficacy to other cART [14–19], their effectiveness in real-life settings has not been fully studied. Effectiveness may be measured by drug tolerability, indicated by switching and adherence, and by HIV RNA suppression.

Here, we describe use of cART classes and regimens overall and as initial therapy in the population-based Multicenter AIDS Cohort Study (MACS) in 2008–2017 and evaluate the effectiveness of commonly used current (2014–2017) regimens by examining their associations with switching, adherence, and suppressed HIV RNA (<20 copies/mL).

## METHODS

### Study Population

In MACS, an ongoing cohort study of men who have sex with men (MSM) [20–22], 7357 men were recruited, including 385 since 2010, in Baltimore/Washington D.C.; Chicago; Los Angeles (LA); Columbus (Ohio); and Pittsburgh. Participants return every 6 months for interviews, physical examinations, and blood collection for concomitant laboratory testing and storage. Study questionnaires are available at https://statepi.jhsph.edu/macs/forms.html. Center-specific institutional review boards approved the protocols, and participants provided informed consent. The cART trend analysis included participants on cART between January 1, 2008, and December 31, 2017. Comparative effectiveness analyses of current regimens, particularly INSTI-cART approved after 2013, included participants on cART during January 1, 2014, to December 31, 2017, and who were seen for at least 2 consecutive study visits (visit-pairs V_i_ and V_i+1_ within 1 year), where the first visit, V_i_, was defined as the index visit.

### cART Regimens

Self-reported use of antiretroviral therapy at each visit was summarized to define cART, according to the United States Department of Health and Human Services Panel guidelines [1]. Protease inhibitor-cART was defined if cART regimens contained a boosted PI; NNRTI-cART if regimens contained a NNRTI without PI; and INSTI-cART if regimens contained an INSTI without PI and NNRTI. Regimens reported by >300 person-visits in 2014–2017 were included in order to have sufficient sample sizes.

### Outcomes

Three outcomes were used to compare the effectiveness of the current regimens: switching, adherence, and HIV-1 RNA suppression (<20 copies/mL). Switching occurred if the regimen reported at the next consecutive visit (V_i+1_) differed from that reported at the index visit (V_i_) or if participants discontinued ART at V_i+1_. A standardized measure of adherence (100% vs <100%) was calculated based on comparing self-reported to prescribed use of each medication in the prior 4 days [23]. To reflect adherence associated with chronic medication use, we required individuals to be on the same regimen for at least 2 consecutive visits (V_i_, V_i+1_) and assessed adherence at V_i+1._ Using the COBAS ApliPrep TaqMan assay (Roche Molecular Systems, Branchburg, NJ) (lower detection limit of 20 copies/mL, available after 2011), HIV RNA plasma levels were measured at each visit [24], and the analyses of HIV RNA suppression at V_i+1_ included only individuals who used the same regimen at V_i_ and V_i+1_ (ie, the same population used for examining adherence). For analyses of suppressed HIV RNA from 2008–2017, we used 50 copies/mL as cutoff to include Roche ultrasensitive test results.

### Covariates

Time-fixed covariates included race/ethnicity (Caucasian [reference], African American, and Hispanic or other race), study site, and time of cohort entry (pre- or post-2001). Time-varying covariates at V_i-1_ (1 visit before V_i_) included age (using a restricted quadratic spline with 4 knots at 20^th^, 40^th^, 60^th^, and 80^th^ percentiles of age [25]), presence of depressive symptoms (a score ≥16 on the Center for Epidemiology Studies-Depression questionnaire), HCV infection (HCV RNA quantified), smoking status (current, past, never [reference]), and use of the following since last visit: alcohol drinking (≥14 (heavy drinking) vs <14 drinks [reference] per week), marijuana, and any use of other recreational drugs (poppers, any cocaine, methamphetamines, heroin, or other street drugs). We also included kidney disease (estimated glomerular filtration rate (eGFR_MDRD_) < 60 mL/min/1.73m^2^ body surface area or proteinuria ≥200 mg/g creatinine), CD4 cell count (<350, 350–499, ≥500 cells/mm^3^ [reference]), and detectable HIV RNA (≥20 vs <20 [reference] copies/mL) at V_i-1._ Gastrointestinal (GI) symptoms (diarrhea, nausea, vomiting, abdominal pain, or bloating) and central nervous system (CNS) sleep-related symptoms (nightmares, vivid dreams, or insomnia) at V_i-1_ used the information about the period since the prior visit. Covariates from V_i-1_ included hypertension (systolic blood pressure ≥ 140 mm Hg, or diastolic ≥ 90 mm Hg, or use of hypertension medication), diabetes (fasting glucose ≥ 126 mg/dL or use of diabetes medication), dyslipidemia (fasting total cholesterol ≥ 200 mg/dL, LDL ≥ 130 mg/dL, HDL <40 mg/dL, triglycerides ≥ 150 mg/dL or use of lipid-lowering medications with self-reported clinical diagnosis), and whether regimens were switched since cART initiation. Other treatment-related variables included calendar year of regimen at V_i_, ART experience before cART initiation, and at V_i-1:_ cumulative years on cART; use of PIs, NNRTIs, and NRTIs; and duration on the regimen reported at V_i-1_.

### Statistical Analysis

Poisson regression with robust variance was used to test for secular trends of regimen use, and to obtain rate ratios when examining associations between regimens and switching, adherence, and HIV-1 RNA suppression. Given that regimens in the MACS were not randomly assigned and prescribing patterns may be affected by treatment history, levels of HIV RNA and CD4 count, and other covariates that prior treatments may affect, we used inverse-probability-of-treatment-and-censoring (IPTC) weighted [26, 27] regression models to control for time-varying confounding when assessing the effect of regimens on switching, adherence, and HIV RNA suppression. Specifically, a longitudinal unordered multinomial logistic model with time-varying regimens (7 regimen categories, with the most reported regimen as the reference category) as the outcome was used to generate stabilized weights. The logistic model for determining the numerator of the weights for regimens included all time-fixed covariates and treatment history variables, including calendar year of current regimen at V_i_. To obtain the denominator of the weights, we also included all time-varying covariates. Similarly, a second logistic model determined the weights of remaining uncensored to control for informative dropout. The final stabilized weights were calculated by multiplying the weights of regimen use and weights of remaining uncensored. We truncated the final stabilized weights at 0.03 (the 1^st^ percentile of overall weights) and 10 (99^th^ percentile) to avoid huge influences by extreme weights [26, 28]. Predictors in the final IPTC-weighted Poisson models included regimen categories, time-fixed covariates, and treatment history at V_i-1_. HIV RNA level ≥200 copies/mL (indicating current virologic failure) at V_i_ also was included in the IPTC-weighted model for switching. We used separate weighted models, including adherence at V_i+1_ for suppressed HIV RNA in the final weighted models. We also age-stratified IPTC-weighted Poisson models for all outcomes by dichotomizing age at 50 years, because some studies suggested that older people may have less immunologic response to ART [29, 30], which may affect clinical practice.

We conducted multiple imputation (MI) for missing data with 25 imputation data sets using multivariable normal models [31], including all three outcomes and all variables used in the generalized logit model to predict the probability of receiving different cART regimens that was used to generate stabilized weights. Specifically, MI imputed missing values for adherence (3% of 3598 person-visits), HIV RNA, HCV, smoking, alcohol drinking, marijuana, other recreational drugs, GI symptoms and CNS sleep-related symptoms, hypertension, depression, kidney disease (6–13%), dyslipidemia (20%), and diabetes (24%). The 25 complete data sets were each analyzed first, and their estimates were combined to calculate average effect estimates and standard errors incorporating the imputation variability [32]. All analyses were conducted with SAS version 9.4 (SAS Institute, Cary, NC).

## RESULTS

### cART Use: 2008–2017

**Figure 1.**
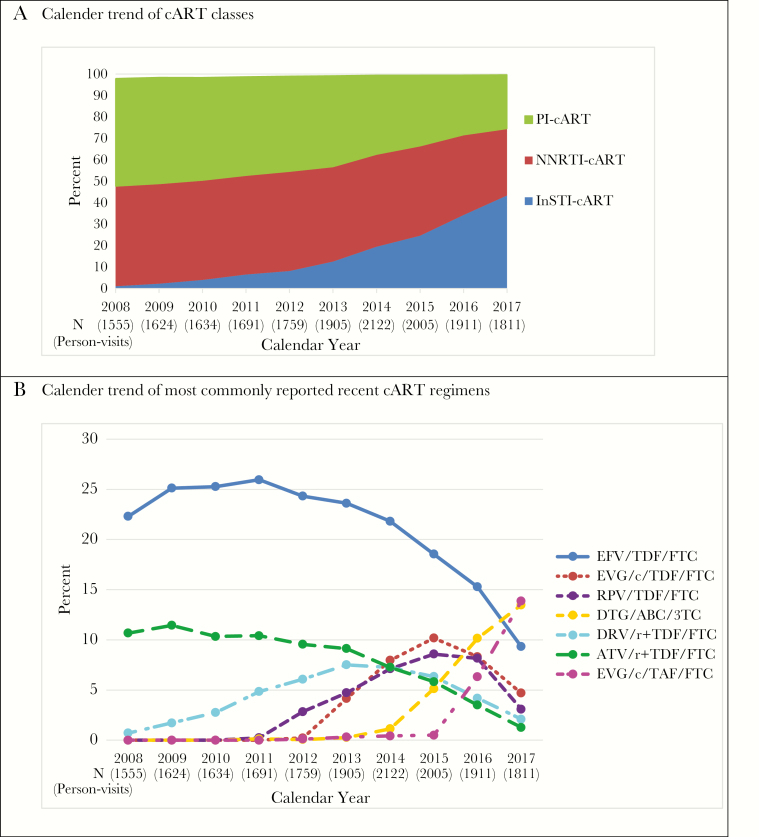
cART Classes and Recent Commonly Used Regimens in the Multicenter AIDS Cohort Study in 2008–2017. A, Calendar trend of cART classes; B, Calendar trend of most commonly reported recent cART regimens.

A total of 1457 MACS participants reported using cART at 18 017 visits (87% of all HIV+ person-visits) in 2008–2017. The median age was 53 years across all visits; 46% were non-Caucasian, 57% were enrolled from 2001 onwards, and 40% started cART before 2000. During 2008–2017, 40% of cART regimens across the person-visits were PI-based and 43% were NNRTI-based. As shown in Figure 1A, from 2008 to 2017, PI-cART use and NNRTI-cART use decreased from 50% to 25%, and 47% to 31%, respectively. Overall, 16% of the person-visits were INSTI-cART-based, which increased from 1% in 2008 to 44% in 2017.

As more INSTI-based regimens have been recently approved, we identified cART regimens commonly used in 2014–2017 (each regimen reported by >300 person-visits overall, Figure 1B). The use of efavirenz/tenofovir disoproxil fumarate/emtricitabine (EFV/TDF/FTC, Atripla) peaked at 26% in 2011 (Figure 1B), but then continuously decreased to 9% in 2017. The use of rilpivirine/TDF/FTC (RPV/TDF/FTC, Complera) and elvitegravir/cobicistat/TDF/FTC (EVG/c/TDF/FTC, Stribild) increased since approval in 2011 and 2012, respectively, and dropped after 2015 when dolutegravir/abacavir/lamivudine (DTG/ABC/3TC, Triumeq) and EVG/c/tenofovir alafenamide/FTC (EVG/c/TAF/FTC, Genvoya) became available. The usage of ritonavir-boosted atazanavir with TDF/FTC (ATV/r+TDF/FTC) dropped from 11% in 2008 to 1% to 2017. Similarly, darunavir/ritonavir+TDF/FTC (DRV/r+TDF/FTC) use continuously dropped from 8% in 2013 to 2% in 2017. Once daily multiclass single-pill regimens increased from 20% in 2008 to 51% in 2017. Percentage of visits with HIV RNA below 50 copies/mL slightly increased from 84% in 2008 to 89% in 2017. In 2017, about 83% cART users had HIV RNA <20 copies/mL.

### Initial cART Regimens

**Figure 2.**
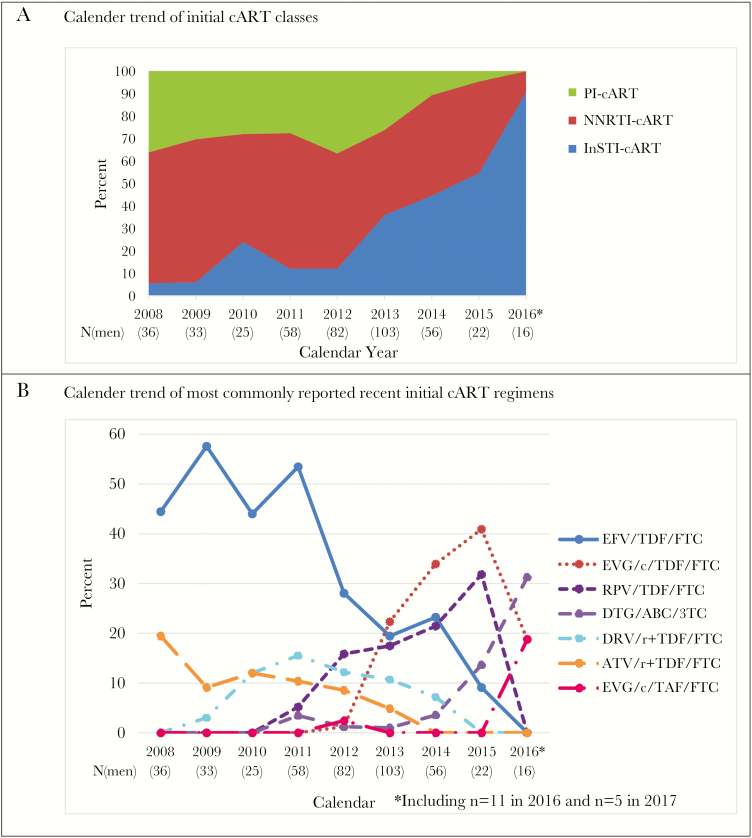
Initial cART Regimens in the Multicenter AIDS Cohort Study in 2008–2017. The last time point 2016* includes 11 observations from 2016 and 5 observations from 2017. A, Calendar trend of initial cART classes; B, Calendar trend of most commonly reported recent initial cART regimens.

In 2008–2017, 431 men initiated cART with median age of 40 years in the MACS. The median (Q1–Q3) nadir CD4 cell count before initiation was 387 (282–537) cells/mm^3^ and increased from 347 (265–437) in 2008–2010 to 533 (369–688) cells/mm^3^ in 2014–2017 (*P* < .001). The median (Q1–Q3) peak HIV RNA level before cART initiation was 54 400 (15 305–157 020) copies/mL overall and decreased from 55 015 (19 293–190 000) in 2008–2010 to 32 307 (4683–127 631) copies/mL in 2014–2017 (*P* = .010). Similar to trends that were observed with overall cART use, percentages of participants on PI- or NNRTI-cART as initial regimens dropped, while the percent on INSTI-cART as initial regimen increased, especially after 2012 (Figure 2A). The use of EFV/TDF/FTC as the first regimen dropped from about 50% from 2008–2009 to 9% in 2015 and 0 in 2016, with increased use of EVG/c/TDF/FTC (1% to 41% from 2012 to 2015) and RPV/TDF/FTC (5% to 32% from 2011 to 2015) until 2015, when new once daily single-pill INSTI-regimens emerged. No one reported using DRV/r+TDF/FTC and ATV/r+TDF/FTC as initial regimens from 2015, and 2014 onwards, respectively. Among 16 men who initiated cART during 2016–2017, 14 initiated with INSTI-cART and 12 used single-pill regimens.

### Comparative Effectiveness of cART Regimens 2014–2017

**Figure 3.**
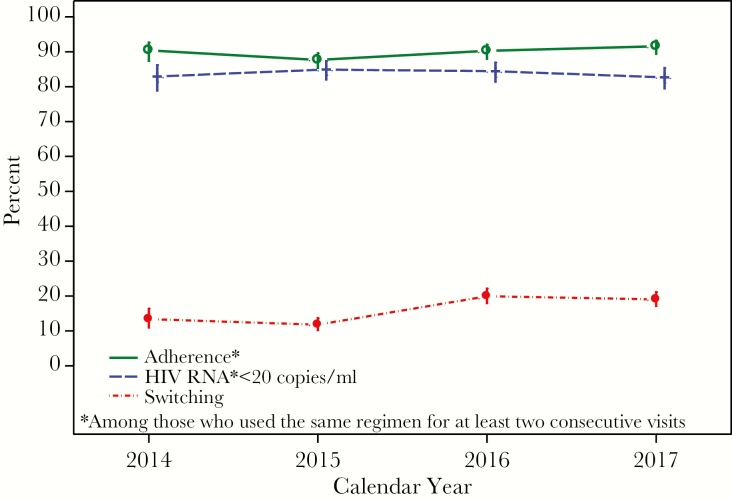
Percentages of Switching cART, Adherence to cART, and Having HIV RNA <20 copies/mL in the Multicenter AIDS Cohort Study in 2014–2017.

In 2014–2017, 729 men reported cART use for at least 2 consecutive visits (<1 year) contributing a total of 3598 paired visits (V_i_, V_i+1_). The 7 most popular regimens reported at V_i_ were as follows: (1) EFV/TDF/FTC (Atripla, n = 1161), (2) EVG/c/TDF/FTC (Stribild, n = 551), (3) RPV/TDF/FTC (Complera, n = 492), (4) DTG/ABC/3TC (Triumeq, n = 401), (5) DRV/r +TDF/FTC (n = 351), (6) ATV/r + TDF/FTC (n = 333), and (7) EVG/c/TAF/FTC (Genvoya, n = 309). Overall, 32% of regimens reported at V_i_ were initial cART regimens. Among those who experienced ART before starting cART, 98% had used NRTI, 46% had used PI, and 21% had used NNRTI. From 2014 to 2017, the overall 16.5% cART switching rate reflected an increase from 13.4% (95% CI, 10.7%, 16.7%) in 2014 to 19.1% (95% CI, 16.9%, 21.5%) in 2017. Adherence to cART was relatively stable at approximately 90%, as was having <20 HIV RNA copies/mL (83–85%) (Figure 3).

To address potential confounding by indication (also known as channeling bias), we examined characteristics at V_i-1_ (the visit prior to V_i_) associated with use of regimens at V_i_ (Table 1). Compared to EFV/TDF/FTC users, more of the DRV/r+TDF/FTC users were Hispanic/other, and fewer EVG/c/TAF/FTC users were African American. These differences, together with differences by study site, hepatitis coinfection, behavioral characteristics, and health status persisted in the multivariable regression models predicting regimen use (Supplemental Table 1).

**Table 1.** Characteristics at V_i-1_ by 7 cART Regimens Reported at V_i_ for 3598 Person-Visits From January 1, 2014 to December 31, 2017

Characteristics Median (Q1–Q3) or %	EFV/TDF/FTC (Atripla)	EVG/c/TDF/FTC (Stribild)	RPV/TDF/FTC (Complera)	DTG/ABC/3TC (Triumeq)	DRV/r + TDF/FTC	ATV/r + TDF/FTC	EVG/c/TAF/FTC (Genvoya)
Number of person-visits	1161	551	492	401	351	333	309
Age (years)	53 (46–59)	48 (34–55)	48 (36–55)	57 (50–64)	48 (38–54)	54 (48–61)	54 (44–61)
Race							
Caucasian	56.6%	50.5%	43.1%	51.9%	27.6%	47.7%	59.5%
African American	27.1%	33.9%	34.3%	34.9%	29.3%	41.4%	21.0%
Hispanic/other	16.3%	15.6%	22.6%	13.2%	43.0%	10.8%	19.4%
Center							
Baltimore/Washington DC	19.5%	29.8%	24.4%	35.7%	12.5%	17.1%	27.8%
Chicago	27.6%	30.7%	15.9%	14.2%	25.1%	25.8%	34.0%
Pittsburgh/Columbus	32.0%	21.2%	32.9%	28.4%	12.3%	35.4%	23.0%
Los Angeles	20.9%	18.3%	26.8%	21.7%	50.1%	21.6%	15.2%
Cohort enrolled after 2001	64.3%	78.0%	78.7%	53.1%	85.2%	50.8%	64.4%
Smoking status							
Current	26.2%	28.2%	28.7%	20.6%	38.5%	29.4%	23.2%
Past	50.6%	36.2%	36.3%	49.9%	28.8%	40.2%	46.0%
Never	23.2%	35.6%	35.0%	29.6%	32.7%	30.4%	30.9%
Drinking ≥14 drinks/week	7.4%	12.4%	5.8%	9.0%	7.7%	8.5%	12.5%
Used recreational drugs	53.4%	63.7%	47.6%	51.9%	49.7%	53.1%	56.7%
Depression	18.4%	30.4%	24.7%	28.0%	35.3%	30.4%	26.1%
HBV-infection	3.9%	2.7%	4.9%	0.5%	3.3%	4.0%	4.2%
HCV-infection	4.6%	3.0%	5.1%	10.3%	6.1%	6.7%	5.7%
Had GI symptoms	18.2%	20.3%	16.5%	23.1%	24.7%	17.1%	20.5%
Had CNS symptoms	45.4%	43.7%	36.5%	41.4%	33.2%	38.3%	48.4%
Hypertension	53.5%	36.6%	38.3%	63.7%	35.0%	42.2%	51.9%
Diabetes	10.3%	7.9%	6.3%	14.7%	6.8%	17.0%	11.6%
Dyslipidemia	77.6%	71.8%	69.4%	87.3%	68.7%	75.3%	77.3%
Kidney disease	17.1%	12.3%	12.9%	31.8%	12.8%	21.9%	18.1%
Experienced ART before cART	31.8%	20.1%	21.7%	45.9%	30.2%	43.5%	31.7%
Calendar year of cART initiation	2004 (1999–2011)	2012 (2001–2014)	201 2(2000–2013)	2002 (1998–2007)	2009 (1999–2012)	2001 (1998–2007)	2005 (1999–2012)
Cumulative years on cART	9.9 (4.4–14.2)	3.0 (1.3–11.1)	3.7 (1.6–11.7)	12.1 (7.2–15.5)	4.9 (2.6–12.6)	11.0 (6.8–14.7)	10.6 (3.8–15.4)
Cumulative years on PI	0.0 (0.0–3.0)	0.0 (0.0–2.5)	0.0 (0.0–3.8)	4.9 (0.0–9.8)	4.0 (2.0–10.3)	11.1 (7.1–14.5)	1.6 (0.0–9.3)
Cumulative years on NNRTI	8.3 (4.0–12.0)	0.1 (0.0–3.0)	2.4 (1.1–4.8)	3.7 (0.0–11.2)	0.0 (0.0–1.7)	0.0 (0.0–2.2)	2.3 (0.0–8.9)
Cumulative years on NRTI	10.7 (4.5–15.7)	3.2 (1.4–12.2)	4.0 (1.8–12.6)	13.7 (7.9–18.8)	5.0 (2.7–15.2)	13.8 (8.0–17.3)	12.0 (3.8–17.3)
Duration of regimen (years)	6.0 (2.7–8.3)	1.1 (0.3–1.9)	1.7 (0.7–2.9)	0.9 (0.3–1.9)	2.2 (0.7–3.8)	4.4 (1.7–7.7)	0.7 (0.3–2.1)
Calendar year of current visit	2015 (2014–2016)	2015 (2014–2016)	2015 (2014–2016)	2016 (2016–2017)	2015 (2014–2016)	2015 (2014–2015)	2017 (2016–2017)
Number of times switched cART regimens							
0	39.3%	45.2%	47.5%	9.8%	34.3%	20.7%	13.3%
1	27.6%	15.9%	18.7%	14.3%	15.7%	18.3%	22.7%
2	15.6%	15.9%	12.2%	19.8%	19.1%	27.0%	21.7%
≥3	17.5%	23.0%	21.6%	56.3%	30.9%	33.9%	42.4%
Current CD4 count (cells/mm^3^)	697 (533–869)	661 (503–882)	707 (547–895)	650 (478–849)	658 (450–853)	669 (520–871)	714 (537–932)
HIV RNA below 20 copies/mL	88.4%	74.4%	80.8%	82.8%	69.6%	77.4%	82.8%

Abbreviations: ABC, abacavir; ATV/r, ritonavir-boosted atazanavir; cART, combination of antiretroviral therapy; CNS, central nervous system sleep related symptoms, including nightmares, vivid dreams, or insomnia; DRV/r, ritonavir-boosted darunavir; DTG, dolutegravir; EFV, efavirenz; EVG/c, cobicistat-boosted elvitegravir; FTC, emtricitabine; GI, gastrointestinal symptoms, including diarrhea, nausea, vomiting, abdominal pain, or bloating; HBV, hepatitis B virus; HCV, hepatitis C virus; NNTRI, Non-Nucleoside Reverse Transcriptase Inhibitors; NRTI, Nucleoside/Nucleotide Reverse Transcriptase Inhibitors; PI, protease inhibitor; Q1, 25^th^ percentile; Q3, 75^th^ percentile; RPV, rilpivirine; TAF, tenofovir alafenamide; TDF, tenofovir disoproxil fumarate; 3TC, lamivudine.

### Regimen Effect on Switching

**Figure 4.**
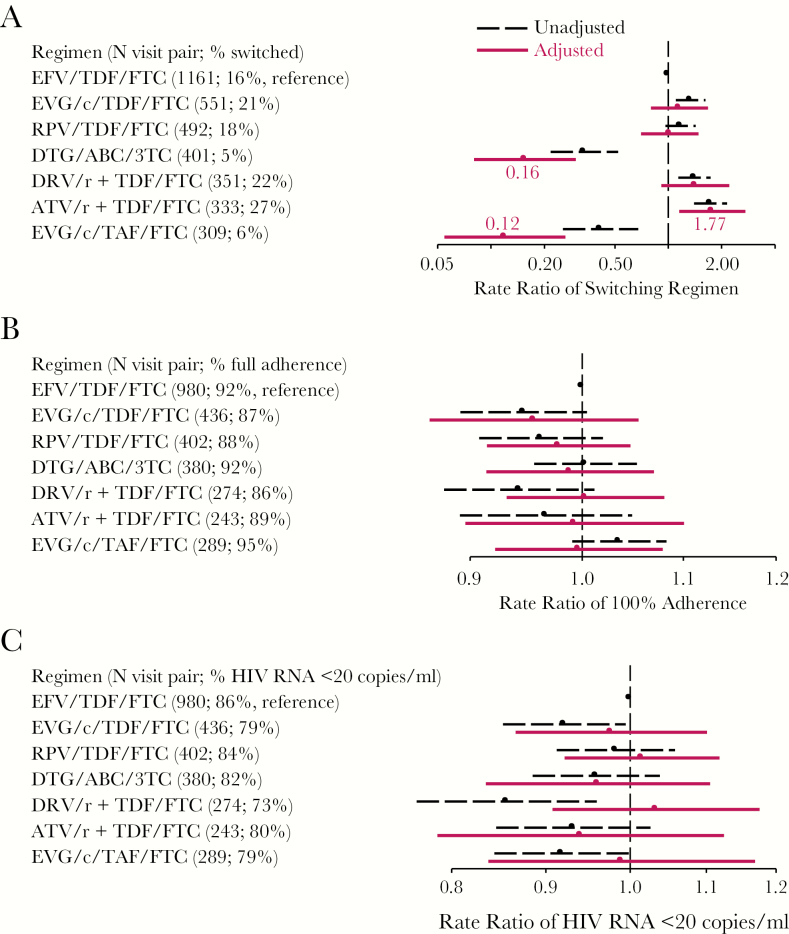
Results from Comparative Effectiveness Analyses of Current Commonly Used Regimens in 2014–2017. A, Rate ratio of switching cART by regimen; B, Rate ratio of 100% adherence to cART by regimen; and, C, Rate ratio of having HIV RNA <20 copies/mL by regimen. Black dots and dashed lines indicate unadjusted rate ratios and 95% confidence intervals, respectively. Red dots and solid lines indicate inverse-probability-of-treatment-and-censoring weighted (adjusted) rate ratios and 95% confidence intervals, respectively. ABC indicates abacavir; ATV/r, ritonavir-boosted atazanavir; DRV/r, ritonavir-boosted darunavir; DTG, dolutegravir; EFV, efavirenz; EVG/c, cobicistat-boosted elvitegravir; FTC, emtricitabine; RPV, rilpivirine; TAF, tenofovir alafenamide; TDF, tenofovir disoproxil fumarate; 3TC, lamivudine.

Switching was highest among ATV/r/TDF/FTC users (27.2%) and lowest among DTG/ABC/3TC users (5.5%); both differed from the 15.2% switching in the EFV/TDF/FTC reference group, which persisted after adjustment for factors associated with their use (Figure 4A). Those using EVG/c/TAF/FTC also were less likely to switch (aRR = 0.12 [95% CI, 0.06, 0.27]). Given DTG/ABC/3TC was approved by U.S. Food and Drug Administration (FDA) in August 2014 and EVG/c/TAF/FTC was approved in November 2015, we had only 2–3 years of observations of these new regimens. In age-stratified analysis, those 50 years and older on PI-based regimens DRV/r+TDF/FTC (aRR = 1.94 [1.23, 3.06]) or ATV/r+TDF/FTC (aRR = 2.13 [1.36, 3.35]) were more likely to switch than the EFV/TDF/FTC users. In younger men, only ATV/r+TDF/FTC users were more likely to switch regimens (aRR = 1.90 [0.87, 4.14]).

We examined self-reported reasons for stopping medications; the majority reported stopping due to prescription changes made by their physicians (Supplemental Table 2). Stopping due to symptoms including CNS sleeping-related problems, such as nightmares/vivid dreams and insomnia/sleeping problems, were mostly reported by EFV/TDF/FTC users. EFV/TDF/FTC, EVG/c/TDF/FTC, DRV/r, or TDF/FTC (Truvada) users reported stopping for increased viral load. Stopping drugs to lower the number of pills or dosages was mostly reported by atazanavir (9%), darunavir (9%), ritonavir (8%) and TDF/FTC (Truvada, 9%) users. Side effects reported by users on INSTI regimens EVG/c/TAF/FTC or DTG/ABC/3TC included nausea/vomiting and fatigue. About 4% reported stopping for personal decisions.

Of the 594 person-visit-pairs with switching, 11% discontinued all ART at V_i+1_, but all resumed cART later during follow-up if V_i+1_ was not the last visit in analysis. Those on EFV/TDF/FTC switched to DTG/ABC/3TC most (18%). Among regimens that were switched, half of EVG/c/TDF/FTC, 19% DTG/ABC/3TC, and 13% ATV/r+TDF/FTC switched to EVG/c/TAF/FTC; 25% of EVG/c/TAF/FTC switched to DTG+ TAF/FTC. Those on RPV/TDF/FTC switched to RPV/TAF/FTC (Odefsey) the most (48%). Those on DRV/r+TDF/FTC switched to DRV/c(Prezcobix)+TAF/FTC (Descovy; 16%) the most.

### Regimen Effect on Adherence and HIV RNA Suppression

We included 3004 visit-pairs with the same regimen at V_i_ and V_i+1_ in analyses of adherence and HIV RNA suppression. Complete adherence ranged from 86% by DRV/r+TDF/FTC users to 95% by EVG/c/TAF/FTC users. After adjustment, adherence did not differ by regimen type (Figure 4B).

Compared to the 86% of EFV/TDF/FTC users with suppressed HIV RNA to <20 copies/mL (Figure 4C), those on EVG/c/TDF/FTC (79%) or DRV/r+/TDF/FTC (73%) or EVG/c/TAF/FTC (79%) had lower rates of HIV RNA suppression overall, but the differences attenuated after adjustment. Among men younger than 50 years, we found that those on EVG/c/TDF/FTC were 15% (95% CI, 2%, 26%) less likely to suppress virus from the IPTC-weighted Poisson model, and in men 50 years and older, those on DRV/r+TDF/FTC had 18% (95% CI, 3%, 34%) higher rates of suppression than EFV/TDF/FTC users. These differences remained even after adjusting for adherence in the final model. We did not observe any differences in percentage change in CD4 cell count by regimen type (data not shown).

## DISCUSSION

We observed substantial decreases in using PI- or NNRTI-based regimens concomitant with a dramatic uptake of INSTI-cARTs overall and as the initial treatment for HIV in 2008–2017. Since 2015, most initiators started with one-pill once-daily INSTI-based regimens DTG/ABC/3TC or EVG/c/TAF/FTC, reflecting the US guidelines’ recommendation [1].

EFV/TDF/FTC was the most commonly reported regimen in 2014–2017, probably because it was the first single-pill multiclass regimen for HIV infection approved by FDA (in July 2006). Prior to 2015, US guidelines recommended it for treatment-naïve patients. However, neuropsychiatric side effects reported by EFV users [33, 34] influenced the guidelines and subsequent practice.

With the recent uptake of new cART classes in clinical practice, it is important to examine their tolerability and effectiveness in real-world settings. We found that among the 7 cART regimens popular in 2014–2017, rates of switching differed. Also, switching by regimen depended on age: lower rates of switching were seen in users of the new INSTI-regimens EVG/c/TAF/FTC or DTG/ABC/3TC and higher rates of switching were observed in users of the multiple-pill regimen ATV/r+TDF/FTC overall, and in DRV/r+TDF/FTC users who were 50 years or older. We observed this difference in the IPTC-weighted models but not in traditional covariate-adjusted models, indicating the importance of appropriately controlling for confounding by indication. Although older men who used DRV/r+TDF/FTC were more likely to switch, those who remained on DRV/r+TDF/FTC had higher rates of HIV RNA <20 copies/ml, indicating that DRV/r+TDF/FTC was highly effective if users could tolerate it. We also observed that older DRV/r+TDF/FTC users had more experience of ART prior to DRV than the EFV/TDF/FTC users (data not shown), which makes sense from a clinical standpoint. Its use by men with more ART experience is consistent with use of darunavir as a salvage regimen. The observation period for new INSTI regimens was relatively short; assessment of switching among users of INSTI should continue. We observed that many who used TDF-containing regimens have switched to regimens containing TAF, reported as incurring less kidney damage than TDF [35–37]. More switching in older TDF/FTC users could be due to perceived safety risks of these drugs in older patients (eg, bone or renal problems). However, long-term effects of new regimens are unknown.

We also found that about half of those who switched regimens reported stopping ART due to prescription changes without further reasons, and 8%–10% of those who stopped ATV, DRV, TDF/FTC, or RTV changed regimens to lower the number of pills. This might reflect the guideline changes and the availability of new approved cART regimens that have strong virologic efficacy, less side effects, and are easier to use [38, 39]. The switching off PI drugs also might reflect a desire to avoid drug interactions with concomitant medications. The economics of medication use (eg, cost and insurance) also may have influenced treatment decisions, but this was beyond the scope of the present study.

All regimens examined in our study had similar effects on adherence, and CD4 count change. An international randomized clinical trial in treatment-naïve women (WAVES) [39] found that EVG/c/TDF/FTC had superior efficacy and lower rate of discontinuation than ATV/r+TDF/FTC. We found that both regimens had similar efficacy in 6 months overall, but about 4% of those who stopped EVG/c/TDF/FTC reported the reason for stopping was increased HIV RNA, which was not reported by ATV/r+TDF/FTC users. When we stratified by age, we found that men younger than 50 years who used EVG/c/TDF/FTC had lower rates of having HIV RNA <20 copies/ml than EFV/TDF/FTC users, which was consistent with our observation of more EVG/c/TDF/FTC users reporting increased HIV RNA as the reason for stopping drug. The different findings in HIV RNA suppression might partially be due to different study populations and study designs, but this warrants further investigation of the effect of long-term use of EVG/c/TDF/FTC on HIV RNA suppression. Our finding of more switching by ATV/r+TDF/FTC users than users of other regimens was consistent with the WAVES’ finding.

The trend of higher CD4 counts at initiation is an indicator of persons living with HIV accessing health care earlier in their infection, as promoted by guidelines and public health programs (https://www.aids.gov/federal-resources/national-hiv-aids-strategy/strategy-implementation/strategy-in-action/). This is highly relevant as early treatment not only improves individual prognosis but also decreases community viral load, and subsequent HIV transmission [40]. Although the study’s MSM population may limit the generalizability of our findings to other populations, MSM comprise the majority (https://www.hiv.gov/hiv-basics/overview/data-and-trends/statistics) of those living with HIV in the US [41]. With the risk group and sociodemographic representation of the recent MACS initiators to that of men living with HIV today in the US [12, 41], our findings are highly encouraging.

These data are highly relevant for chronically-infected users of cART. Whereas current clinical trials usually include treatment-naïve individuals, we were able to examine regimen uptake and effectiveness more broadly. In this analysis, 31% of our study sample were on their first cART regimens so more than two-thirds were treatment-experienced.

In summary, most recent cART initiators started with INSTI-containing single-pill regimens, consistent with guidelines. Findings indicate that factors beyond those studied here, such as the need for salvage therapy, unique personal characteristics, drug interactions, and cost, may influence treatment decisions.

## Supplementary Data

Supplementary materials are available at *Open Forum Infectious Diseases* online. Consisting of data provided by the authors to benefit the reader, the posted materials are not copyedited and are the sole responsibility of the authors, so questions or comments should be addressed to the corresponding author.

ofz333_suppl_supplementary_table_s1

ofz333_suppl_supplementary_table_s2
