# Supplementary material for: Recent Trends and Effectiveness of Antiretroviral Regimens Among Men Who Have Sex With Men Living With HIV in the United States: The Multicenter AIDS Cohort Study (MACS) 2008–2017
Source: Open Forum Infect Dis. 2019 Jul 16;6(9):ofz333. doi: 10.1093/ofid/ofz333 (PMC6798255; doi:10.1093/ofid/ofz333)
Supplement: ofz333_suppl_supplementary_table_s1 [file ofz333_suppl_supplementary_table_s1.docx]

Supplemental Table 1. Predictors of regimen use with EFV/TDF/FTC (n=1161 person-visits) as reference regimen

| *Predictors* | *EVG/c/TDF/FTC (n=551) OR (95% CI)* | *RPV/TDF/FTC (n=492) OR (95% CI)* | *DTG/ABC/LAM (n=401) OR (95% CI)* | *DRV/r+TDF/FTC (n=351) OR (95% CI)* | *ATV/r+TDF/FTC (n=333) OR (95% CI)* | *EVG/c/TAF/FTC (n=309) OR (95% CI)* |
| --- | --- | --- | --- | --- | --- | --- |
| Age*, per 1 year | 0.97 (0.94,1.01) | 0.98 (0.94,1.01) | 1.02 (0.96,1.08) | 0.97 (0.93,1.01) | 0.98 (0.93,1.03) | 0.97 (0.92,1.02) |
| _Age# | 0.99 (0.88,1.12) | 1.07 (0.96,1.21) | 0.90 (0.76,1.07) | 1.04 (0.90,1.21) | 0.96 (0.81,1.15) | 1.06 (0.89,1.26) |
| __Age# | 1.43 (0.87,2.35) | 0.82 (0.51,1.32) | 2.02 (1.1,3.71) | 1.36 (0.74,2.51) | 1.41 (0.71,2.77) | 1.14 (0.60,2.18) |
| ___Age# | 0.49 (0.25,0.95) | 1.23 (0.67,2.27) | 0.38 (0.19,0.79) | 0.53 (0.23,1.19) | 0.74 (0.32,1.71) | 0.72 (0.33,1.58) |
| Race, Black vs. White | 0.71 (0.50,0.99) | 1.06 (0.75,1.49) | 1.26 (0.84,1.9) | 0.77 (0.49,1.22) | 1.50 (0.91,2.48) | 0.40 (0.25,0.63) |
| Race, Hispanic vs. White | 0.66 (0.43,1.01) | 1.30 (0.85,1.97) | 1.23 (0.73,2.09) | 1.92 (1.16,3.19) | 0.84 (0.43,1.62) | 1.04 (0.61,1.79) |
| Site, Baltimore / Washington DC vs. LA | 2.05 (1.37,3.08) | 1.24 (0.84,1.83) | 2.58 (1.64,4.05) | 0.40 (0.24,0.66) | 0.70 (0.39,1.25) | 2.87 (1.67,4.92) |
| Site, Chicago vs. LA | 1.74 (1.15,2.63) | 0.62 (0.41,0.94) | 0.57 (0.34,0.96) | 0.76 (0.47,1.22) | 0.56 (0.32,1) | 2.89 (1.69,4.94) |
| Site, Pittsburgh / Columbus vs. LA | 0.58 (0.38,0.89) | 0.80 (0.54,1.19) | 0.94 (0.58,1.51) | 0.18 (0.10,0.30) | 0.48 (0.27,0.84) | 0.84 (0.48,1.49) |
| Cohorts post-2001 vs. pre-2001 | 1.01 (0.65,1.56) | 0.96 (0.64,1.44) | 0.92 (0.58,1.46) | 4.08 (2.21,7.53) | 0.93 (0.52,1.69) | 1.07 (0.64,1.77) |
| Current smoker* vs. non-smoker | 0.54 (0.37,0.77) | 0.69 (0.48,0.97) | 0.52 (0.33,0.81) | 1.23 (0.80,1.89) | 1.23 (0.73,2.07) | 0.65 (0.40,1.05) |
| Past smoker* vs. non-smoker | 0.53 (0.38,0.72) | 0.51 (0.38,0.7) | 0.71 (0.49,1.03) | 0.40 (0.27,0.60) | 0.48 (0.30,0.77) | 0.70(0.47,1.05) |
| Alcohol* >=14 vs. <14 drinks/week | 1.97 (1.20,3.24) | 0.99 (0.57,1.69) | 1.11 (0.62,2.00) | 1.30 (0.67,2.50) | 1.02 (0.49,2.14) | 1.55 (0.83,2.88) |
| Used marijuana* | 1.12 (0.82,1.51) | 0.52 (0.39,0.71) | 0.89 (0.63,1.26) | 0.63 (0.43,0.92) | 1.00 (0.65,1.53) | 0.62 (0.41,0.92) |
| Used other recreational drugs* | 1.06 (0.80,1.40) | 0.75 (0.56,1.01) | 0.88 (0.63,1.23) | 0.94 (0.65,1.36) | 1.31 (0.86,2.02) | 1.25 (0.86,1.82) |
| Depressed* | 1.72 (1.22,2.42) | 1.35 (0.96,1.91) | 1.64 (1.10,2.44) | 2.53 (1.67,3.84) | 2.73 (1.72,4.33) | 1.34 (0.86,2.07) |
| HCV-infection* | 0.32 (0.16,0.65) | 0.46 (0.25,0.86) | 0.55 (0.29,1.05) | 0.17 (0.08,0.37) | 0.21 (0.09,0.46) | 0.43 (0.20,0.95) |
| Had GI symptoms* | 1.04 (0.72,1.49) | 0.98 (0.69,1.41) | 1.95 (1.29,2.95) | 1.24 (0.81,1.9) | 0.81 (0.49,1.36) | 1.64 (1.03,2.61) |
| Had CNS symptoms* | 0.81 (0.60,1.1) | 0.69 (0.52,0.91) | 0.74 (0.53,1.03) | 0.46 (0.31,0.68) | 0.72 (0.48,1.10) | 1.01 (0.69,1.47) |
| Hypertension* | 0.67 (0.49,0.91) | 0.72 (0.54,0.96) | 1.03 (0.72,1.46) | 0.62 (0.42,0.91) | 0.44 (0.29,0.68) | 0.91 (0.60,1.37) |
| Diabetes* | 0.50 (0.28,0.90) | 0.67 (0.40,1.11) | 0.58 (0.33,1.01) | 0.40 (0.21,0.78) | 1.39 (0.73,2.63) | 0.47 (0.24,0.92) |
| Dyslipidemia* | 0.96 (0.68,1.37) | 0.80 (0.58,1.11) | 1.31 (0.84,2.05) | 0.85 (0.56,1.29) | 1.06 (0.65,1.75) | 0.72 (0.45,1.17) |
| Kidney disease* | 0.97 (0.64,1.48) | 0.76 (0.51,1.13) | 1.63 (1.08,2.46) | 0.92 (0.56,1.52) | 0.75 (0.44,1.29) | 1.01 (0.62,1.64) |
| Experienced ART before cART | 1.09 (0.68,1.76) | 1.20 (0.79,1.84) | 1.11 (0.69,1.78) | 1.68 (0.97,2.91) | 1.14 (0.64,2.03) | 0.72 (0.42,1.23) |
| Cumulative years on cART*, per year | 1.1 0 (1.01,1.20) | 1.08 (1.00,1.16) | 1.03 (0.95,1.12) | 0.93 (0.84,1.02) | 0.83 (0.75,0.91) | 0.99 (0.91,1.08) |
| Cumulative years on PI*, per year | 0.95 (0.89,1.01) | 1.12 (1.06,1.18) | 1.05 (0.99,1.11) | 1.27 (1.17,1.37) | 1.74 (1.6,1.89) | 1.09 (1.03,1.17) |
| Cumulative years on NNRTI*, per year | 0.71 (0.67,0.76) | 0.91 (0.86,0.96) | 0.80 (0.75,0.84) | 0.55 (0.5,0.61) | 0.66 (0.6,0.72) | 0.79 (0.74,0.84) |
| Cumulative years on NRTI*, per year | 1.12 (1.03,1.22) | 0.97 (0.90,1.04) | 1.12 (1.03,1.21) | 1.09 (0.99,1.20) | 0.96 (0.87,1.05) | 1.11 (1.02,1.20) |
| Duration of regimen*, per year | 0.58 (0.54,0.62) | 0.67 (0.63,0.71) | 0.62 (0.58,0.66) | 0.92 (0.85,0.99) | 1.11 (1.02,1.20) | 0.64 (0.59,0.69) |
| Calendar year of current regimen, per year | 1.59 (1.38,1.82) | 1.50 (1.31,1.72) | 4.47 (3.75,5.33) | 0.96 (0.8,1.15) | 0.65 (0.53,0.81) | 7.76 (6.22,9.67) |
| Ever switched cART regimen* | 1.41 (0.95,2.1) | 0.95 (0.64,1.40) | 3.36 (1.99,5.66) | 2.93 (1.82,4.74) | 4.31 (2.45,7.58) | 4.56 (2.66,7.83) |
| CD4 count* 350-499 vs. >=500 cells/ul | 1.03 (0.70,1.53) | 0.60 (0.40,0.90) | 1.13 (0.73,1.75) | 0.73 (0.44,1.19) | 0.77 (0.44,1.35) | 0.59 (0.34,1.03) |
| CD4 count* <350 vs. >=500 cells/ul | 1.05 (0.59,1.89) | 1.14 (0.67,1.92) | 1.39 (0.75,2.58) | 1.55 (0.87,2.78) | 1.05 (0.50,2.18) | 0.83 (0.41,1.69) |
| HIV RNA >=200 vs. <200 copies/ml | 1.24 (0.76,2.04) | 1.22 (0.76,1.96) | 1.38 (0.74,2.59) | 1.03 (0.59,1.8) | 0.87 (0.45,1.72) | 1.49 (0.73,3.02) |

ABC = abacavir; ATV/r = ritonavir-boosted atazanavir; DRV/r = ritonavir-boosted darunavir; DTG = dolutegravir; EFV = efavirenz; EVG/c = cobicistat-boosted elvitegravir; FTC = emtricitabine; RPV = rilpivirine; TAF=Tenofovir alafenamide; TDF = tenofovir disoproxil fumarate; 3TC = lamivudine; OR = odds ratio; CI = confidence interval; *At the visit prior to the current regimen; #Restricted quadratic spline basis function variables for age; LA = Los Angeles; GI = gastrointestinal symptoms, including diarrhea, nausea, vomiting, abdominal pain or bloating; CNS = central nervous system sleep related symptoms, including nightmares, vivid dreams or insomnia. cART= combination of antiretroviral therapy; PI = Protease Inhibitors; NNTRI = Non-Nucleoside Reverse Transcriptase Inhibitors; NRTI = Nucleoside/Nucleotide Reverse Transcriptase Inhibitors.
