# Supplementary material for: Recent Trends and Effectiveness of Antiretroviral Regimens Among Men Who Have Sex With Men Living With HIV in the United States: The Multicenter AIDS Cohort Study (MACS) 2008–2017
Source: Open Forum Infect Dis. 2019 Jul 16;6(9):ofz333. doi: 10.1093/ofid/ofz333 (PMC6798255; doi:10.1093/ofid/ofz333)
Supplement: ofz333_suppl_supplementary_table_s2 [file ofz333_suppl_supplementary_table_s2.docx]

Supplemental Table 2. Reasons for stopping antiretroviral drugs in 2014-2017

| *Side effects N(%)* | *Atripla (EFV/TDF/FTC)* | *Atazanavir* | *Complera (RPV/TDF/FTC)* | *Darunavir* | *Genvoya (EVG/c/TAF/FTC)* | *Ritonavir* | *Stribild (EVG/c/TDF/FTC)* | *Truvada (TDF/FTC)* | *Triumeq (DTG/ABC/3TC)* |
| --- | --- | --- | --- | --- | --- | --- | --- | --- | --- |
| N (person-visits) | 124 | 53 | 62 | 54 | 12 | 98 | 75 | 98 | 14 |
| Dizziness/ headaches | 2 (1.6) | 1 (1.9) | 0 (0.0) | 0 (0.0) | 0 (0.0) | 1 (1.0) | 0 (0.0) | 1 (1.0) | 0 (0.0) |
| Nausea/ vomiting | 1 (0.8) | 1 (1.9) | 0 (0.0) | 0 (0.0) | 1 (8.3) | 2 (2.0) | 0 (0.0) | 1 (1.0) | 2 (14.3) |
| Abdominal pain/ pancreatitis/ bloating/ cramps | 0 (0.0) | 1 (1.9) | 0 (0.0) | 0 (0.0) | 0 (0.0) | 1 (1.0) | 0 (0.0) | 1 (1.0) | 0 (0.0) |
| Diarrhea | 0 (0.0) | 1 (1.9) | 0 (0.0) | 1 (1.9) | 0 (0.0) | 1 (1.0) | 1 (1.3) | 2 (2.0) | 0 (0.0) |
| kidney stones | 0 (0.0) | 1 (1.9) | 0 (0.0) | 0 (0.0) | 0 (0.0) | 1 (1.0) | 1 (1.3) | 1 (1.0) | 0 (0.0) |
| High cholesterol/ high triglycerides | 1 (0.8) | 0 (0.0) | 0 (0.0) | 0 (0.0) | 0 (0.0) | 1 (1.0) | 0 (0.0) | 1 (1.0) | 0 (0.0) |
| Nightmares/ vivid dreams | 3 (2.4) | 1 (1.9) | 1 (1.6) | 0 (0.0) | 0 (0.0) | 1 (1.0) | 0 (0.0) | 1 (1.0) | 0 (0.0) |
| Insomnia/ sleeping problems | 3 (2.4) | 0 (0.0) | 0 (0.0) | 0 (0.0) | 0 (0.0) | 0 (0.0) | 0 (0.0) | 1 (1.0) | 0( 0.0) |
| Fatigue | 4 (3.2) | 1 (1.9) | 0 (0.0) | 0 (0.0) | 2 (16.7) | 2 (2.0) | 1 (1.3) | 1 (1.0) | 1 (7.1) |
| Increased viral load | 4 (3.2) | 0 (0.0) | 0 (0.0) | 1 (1.9) | 0 (0.0) | 1 (1.0) | 3 (4.0) | 1 (1.0) | 1 (7.1) |
| Personal decision | 3 (2.4) | 1 (1.9) | 4 (6.5) | 3 (5.6) | 0 (0.0) | 4 (4.1) | 4 (5.3) | 4 (4.1) | 2 (14.3) |
| Prescription changes by physician | 65 (52.4) | 29 (54.7) | 31 (50) | 25 (46.3) | 5 (41.7) | 48 (49.0) | 38 (50.7) | 49 (50.0) | 10 (71.4) |
| Inconvenient/ran out/vacation | 0 (0.0) | 3 (5.7) | 4 (6.5) | 5 (9.3) | 0 (0.0) | 8 (8.2) | 4 (5.3) | 5 (5.1) | 0 (0.0) |
| Changed drug to lower #pills/dosing | 3 (2.4) | 5 (9.4) | 1 (1.6) | 5 (9.3) | 0 (0.0) | 8 (8.2) | 1 (1.3) | 9 (9.2) | 0 (0.0) |

ABC = abacavir; DTG = dolutegravir; EFV = efavirenz; EVG/c = cobicistat-boosted elvitegravir; FTC = emtricitabine; RPV = rilpivirine; TAF=Tenofovir alafenamide; TDF = tenofovir disoproxil fumarate; 3TC = lamivudine
